# Comparison study of two anastomosis techniques in right hemicolectomy: a systematic review and pooling up analysis

**DOI:** 10.1007/s00384-025-04835-8

**Published:** 2025-02-25

**Authors:** Xiao-Qiang Zhang, Run-xi Tang, Chao-Fu Zhang, Ming-Yang Xia, Lei-Yuan Shuai, Hua Tang, Guang-Yan Ji

**Affiliations:** 1 Department of Gastrointestinal Surgery, The First Affiliated Hospital of Chongqing Medical University, No.1 Youyi Road, Yuanjiagang District, Chongqing, 400016 China; 2 Shanxi Provincial Institute of Traditional Chinese Medicine, Shanxi, 030021 China; 3 Department of Anorectal Surgery, Jiangjin Central Hospital of Chongqing, Chongqing, 404000 China

**Keywords:** Right hemicolectomy, Anastomosis, Postoperative complications, Side-to-side, End-to-side

## Abstract

**Purpose:**

This study aims to compare side-to-side anastomosis (SSA) and end-to-side anastomosis (ESA) in laparoscopic right hemicolectomy from multiple perspectives to guide the selection of the optimal anastomotic technique.

**Methods:**

This review was pre-registered with PROSPERO (CRD42024614418). A comprehensive literature search was performed using Embase, PubMed, Cochrane Library, and China Biology Medicine (CBM). The primary outcome was anastomotic complications, and secondary outcomes included non-anastomotic complications, short-term prognosis, and surgical parameters.

**Results:**

A total of 18 articles involving 14,555 participants were included in this systematic review and meta-analysis. No significant difference was found between SSA and ESA regarding overall anastomotic complications (OR = 1.14, 95% CI = 0.81 to 1.62, *P* = 0.45). However, SSA showed advantages in reducing postoperative anastomotic bleeding (OR = 0.64, 95% CI = 0.45 to 0.90, *P* = 0.01), while ESA appeared more favorable for reducing anastomotic leakage (AL) (OR = 1.29, 95% CI = 0.97 to 1.73, *P* = 0.08) and intestinal obstruction (OR = 1.20, 95% CI = 0.99 to 1.47, *P* = 0.07), though these differences were not statistically significant. No significant differences were found in non-anastomotic complications, short-term prognosis, or surgical parameters.

**Conclusion:**

Current clinical evidence suggests that SSA is more effective than ESA in reducing postoperative anastomotic bleeding during right hemicolectomy for cancer. However, no significant differences were observed between the two techniques regarding overall anastomotic.

**Supplementary Information:**

The online version contains supplementary material available at 10.1007/s00384-025-04835-8.

## Introduction

Right-sided colon cancer is a common malignancy of the digestive tract. Laparoscopic right hemicolectomy has gained widespread clinical acceptance due to its minimally invasive nature and ability to facilitate faster recovery [1, 2]. In this procedure, the choice of anastomotic technique can influence postoperative bowel function and quality of life [3, 4]. Two commonly used anastomotic techniques are SSA and ESA.

**Fig. 1:**
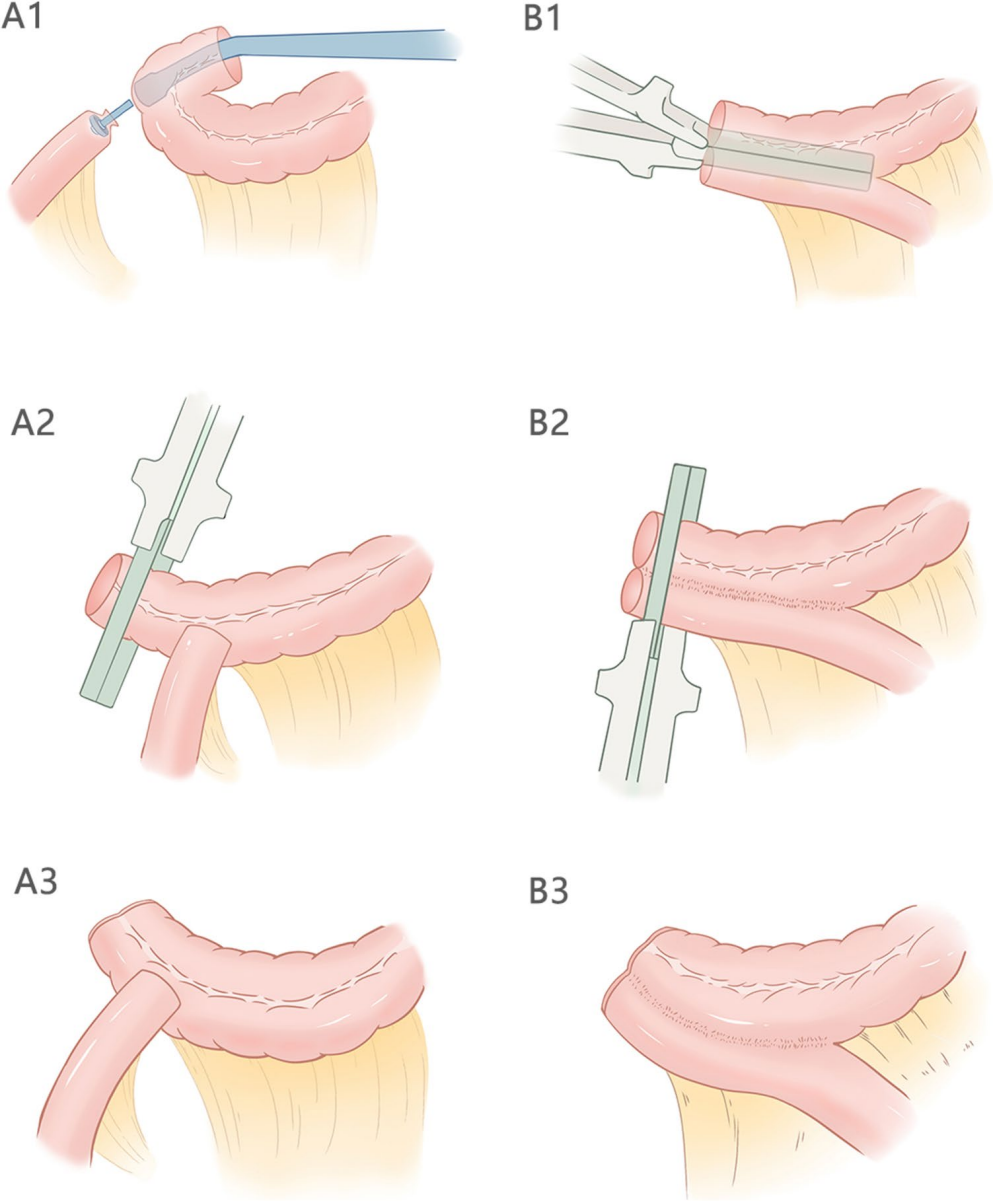
Schematic diagram of two anastomosis modes. **A** End-side anastomosis. **B** Side-side anastomosis

ESA involves connecting the end of the ileum to the side of the remaining colon. This technique mimics natural intestinal flow and is preferred for its anatomical consistency [5]. Several studies suggest ESA may lower anastomotic leakage rates and promote faster recovery, though it carries a higher risk of anastomotic stricture due to a narrower anastomotic site [6–8]. SSA, on the other hand, creates a wider, parallel connection between the ileum and colon, which may reduce the risk of stricture and improve postoperative bowel function [9] (Fig. 1).

The choice between ESA and SSA depends on multiple factors, including patient anatomy, surgeon experience, and specific clinical circumstances [5]. Current literature does not clearly demonstrate the superiority of one technique over the other [6, 8]. Therefore, this study aims to conduct a meta-analysis to further explore differences in postoperative outcomes between SSA and ESA.

## Methods

This systematic review was conducted in accordance with the PRISMA Checklist (Supplementary Information) and was pre-registered with PROSPERO (CRD:42024614418). The link is https://www.crd.york.ac.uk/prospero/display_record.php?ID=CRD42024614418.

### Inclusion and exclusion criteria

The studies included in this meta-analysis were performed according to the PICOS principle, with the inclusion criteria being patients undergoing right hemicolectomy due to colon cancer (P); the exposure factor were anastomosis methods (I); ESA and SSA were compared (C); postoperative anastomotic complications, non-anastomotic complications, short-term prognosis and surgical status (O); the types of the study were the Randomized Controlled Trial (RCT) and cohort studies (S); the surgical method is limited to laparoscopic surgery (including laparoscopic-assisted surgery and total laparoscopic surgery). There are no restrictions on language, sample size, and date of publication.

The exclusion criteria were as follows: (1) patients undergoing surgery for Crohn’s disease or non-intestinal tumors; (2) patients who underwent traditional laparotomy or were temporarily converted to laparotomy during surgery; (3) the study is divided into case reports, single-arm studies, letters to editors, summaries, and conference abstracts; (4) research with incomplete data; and duplicate data (when two studies contain overlapping data, the study with the larger sample size will be included).

### Search strategy

The databases searched included Embase, Cochrane library, PubMed, and CBM. The “Colectomy” search terms utilized were “Colectomy” OR “Large Bowel Resection” OR “Hemicolectomy” OR “Resection, Large Bowel”; The “Anastomosis” search terms are: “Anastomosis” OR “Anastomosis, Surgical” OR “Surgical Anastomoses” OR “end‑to‑side” AND “side‑to‑side”; the search was conducted until December 2024.

### Data extraction

The baseline data extracted from each study included the first author, year, country, study design, number of patients, age, sex, body mass index (BMI), American Society of Anesthesiologists (ASA) grade, and diabetes mellitus (DM). Tumor-related information was also collected, including tumor location, pathological stage, and tumor size. Data on postoperative outcomes were gathered. The selected articles were independently evaluated by two researchers who conducted the data extraction, both of whom received training before starting their work. Any discrepancies arising during the process were resolved through consultation.

### Assessment of risk of bias in included studies

Risk of bias assessment RCTs were assessed using the Jadad scale, while cohort studies were evaluated using the Newcastle–Ottawa Scale (NOS). Bias was assessed using the Cochrane Collaboration tool, with studies scoring < 7 on the Newcastle–Ottawa Quality Assessment Scale (NOS) considered at risk of bias. A third investigator adjudicating when there was a large discrepancy in the ratings. Specific scoring details of the included studies can be found in the Supplementary Materials.

To enhance the robustness of our conclusions, we conducted subgroup analyses based on study type (cohort studies and RCTs). At the same time, given that most of the included studies performed in extracorporeal anastomosis (ECA) for SSA and ESA, we again performed subgroup analyses to specifically examine anastomotic complications in ECA cases.

### Statistical analysis

Primary outcomes included overall anastomotic complications (leakage, bleeding, obstruction). Secondary outcomes included non-anastomotic complications (wound infection, mortality); postoperative recovery (length of hospital stay, time to first gas, tolerable diet); and surgical parameters (operative duration, blood loss).

In the current meta-analysis, continuous variables are presented as the mean and standard deviation (SD), and categorical variables are presented as proportions. For dichotomous and continuous variables, odds ratios (ORs) and mean differences (MDs) were calculated, and 95% confidence intervals (CIs) were calculated. The *I*^2^ value and the results of the chi-squared test were used to assess the statistical heterogeneity. High heterogeneity was considered when *I*^2^ > 50%; in such cases, the random effects model was used, and *P* < 0.1 was considered statistically significant. The fixed effects model was used when *I*^2^ ≤ 50%, and *P* < 0.05 was considered statistically significant. Funnel plots were used to check for potential publication bias in the outcome measures. When included studies reported continuous data in the form of median (range) or median (IQR, interquartile range), the mean (SD) was calculated using the methods suggested by Hozo et al. and Wan et al. [10, 11]. This meta-analysis was performed with RevMan 5.3 (the Cochrane Collaboration, London, UK).

## Results

### Study characteristics and baseline information

**Fig. 2:**
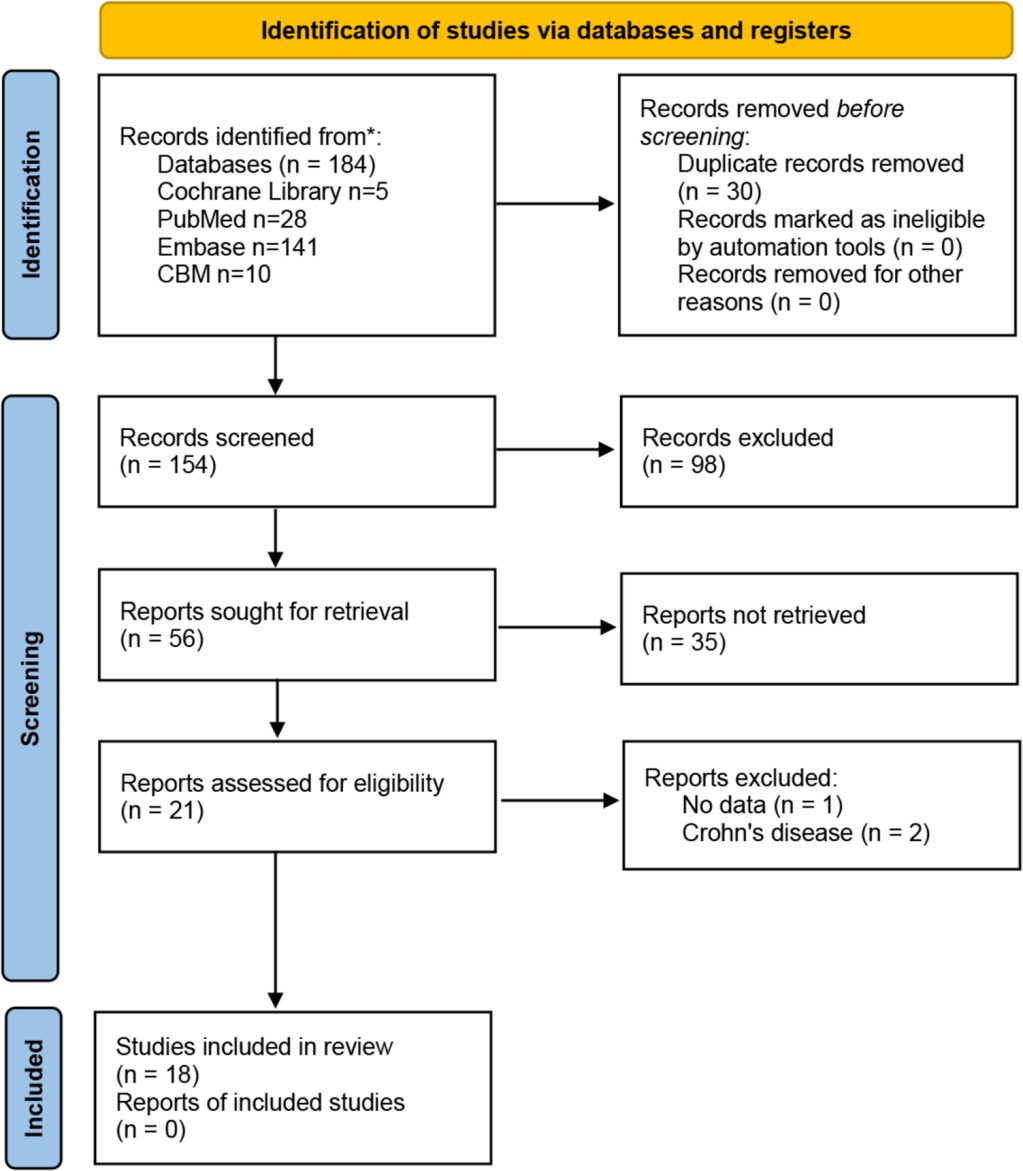
PRISMA 2020 study selection flow diagram

In the primary search, a total of 183 studies were identified (27 in PubMed, 141 in Embase, 10 in CBM, and 5 in the Cochrane Library) and 30 studies were screened after the exclusion of duplicate records. Titles and abstracts were screened, and 20 studies were left for full-text assessment. After excluding three articles, we added one more study. Finally, a total of 18 studies compared surgical outcomes between SSA and ESA [4–9, 12–23] (Fig. 2).

A total of 18 studies including 14,555 patients were included in this meta-analysis. The publication years ranged from 1993 to 2024, and the study dates ranged from 1981 to 2022. There were 16 retrospective studies, two randomized controlled trial (RCT) [4, 5]. Three studies were conducted in Australia, three studies were conducted in South Korea, one study was conducted in Denmark, one study was conducted in Singapore, one study was conducted in France, one study was conducted in Italy, and one study was conducted in Spain. Seven studies were conducted in China. The sample size and the scores of the NOS of each study are shown in Table 1. The quality of randomized controlled studies is assessed in Table 2.

**Table 1:** Characteristics of the studies included in the meta-analysis

Author	Year	Country	Study date	Patients	Study type	Study design	Language	Approach	NOS score
Rajagopalan, A	2023	Australia	2007–2021	6921	Retrospective	Multi-center	English	ECA	8
Würtz, H. J	2022	Denmark	2015–2019	1196	Retrospective	Multi-center	English	ECA and ICA	8
Rajan, R	2022	Australia	2008–2020	1125	Retrospective	Multi-center	English	ECA	7
Baqar, A. R	2022	Australia	2010–2020	1040	Retrospective	Multi-center	English	ECA	9
Xia, T	2021	China	2017–2018	92	Retrospective	Single center	English	ECA	7
Lin, S. Y	2022	Singapore	2016–2019	194	Retrospective	Single center	English	ECA	7
Lee, K. H	2016	South Korea	2009–2012	89	Retrospective	Single center	English	NA	7
Kim,M. H	2022	South Korea	2016–2019	130	RCT	Single center	English	ECA	NA
Puleo, S	2013	Italy	2002–2007	944	Retrospective	Multi-center	English	NA	6
Golda, T	2013	Spain	2006–2011	176	Retrospective	Single center	English	NA	9
Kracht, M	1993	France	1981–1990	279	RCT	Multi-center	English	NA	NA
Yao Mingquan	2023	China	2020–2022	62	Retrospective	Single center	Chinese	ICA	7
Wang Maofeng	2022	China	2019–2021	79	Retrospective	Single center	Chinese	ECA and ICA	7
Huang Zudong	2020	China	2014–2018	365	Retrospective	Single center	Chinese	ECA	7
Cheng Kangwen	2019	China	2015–2018	89	Retrospective	Single center	Chinese	ECA	7
Li Fang-kun	2018	China	2014–2017	193	Retrospective	Single center	Chinese	ECA	9
Zheng liu	2014	China	2009–2012	379	Retrospective	Single center	English	ECA	9
Seijong Kim	2024	South Korea	2007–2016	1202	Retrospective	Single center	English	NA	9

Abbreviations: *NA* measurement not available, *NOS* Newcastle–Ottawa Scale, *RCT* randomized controlled trial, *ECA* extracorporeal anastomosis, *ICA* intracorporeal anastomosis

**Table 2:** Risk-of-bias assessment of included RCTs

Reference	Random sequence generation (selection bias)	Allocation concealment (selection bias)	Blinding of patients (performance bias)	Blinding of outcome assessment (detection bias)	Incomplete outcome data (attrition bias)	Selective reporting (reporting bias)	Other bias	Overall risk of bias
Kim, M. H. et al	+	+	+	+	+	+	+	Low
Kracht, M. et al	+	+	+	+	+	+	+	Low

The baseline information, including age, sex, BMI, American Society of Anesthesia (ASA) and surgical methods, was pooled, and no differences were found between the SSA and the ESA. The summary meta-analysis of baseline information in each study is shown in Table 3.

**Table 3:** Summary of characteristics between SSA and ESA

Characteristics	Studies	Participants (SSA/ESA)	MD/OR (95% Cl)	Model	Heterogeneity
*Basic information of patients*					
Age	11	2279/1256	− 0.38 [− 1.39, 0.63]; *P* = 0.46	RE	*I*^2^ = 60%; *P* = 0.006
Male	15	9151/3321	0.95 [0.90, 1.06]; *P* = 0.55	FE	*I*^*2*^ = 32%; *P* = 0.11
BMI	9	1116/852	0.04 [−0.34, 0.42]; *P* = 0.82	RE	*I*^2^ = 56%; *P* = 0.02
DM	6	1286/1021	1.12 [0.91, 1.39]; *P* = 0.29	FE	*I*^2^ = 0%; *P* = 0.94
ASA					
1	8	7564/2521	1.20 [0.85, 1.69]; *P* = 0.3	RE	*I*^2^ = 63%; *P* = 0.009
2	8	7564/2521	1.11 [1.01, 1.22]; *P* = 0.03	FE	*I*^2^ = 0%; *P* = 0.75
3	7	6368/2330	0.77 [0.59, 1.02]; *P* = 0.06	RE	*I*^2^ = 55%; *P* = 0.04
4	3	6195/2116	1.55 [0.71, 3.38]; *P* = 0.27	RE	*I*^2^ = 75%; *P* = 0.02
*Surgery-related information*					
Tumor site					
Appendix and caecum	6	6717/1913	0.91 [0.82, 1.02]; *P* = 0.10	FE	*I*^2^ = 7%; *P* = 0.37
Ascending colon	6	6717/1913	1.04 [0.93, 1.15]; *P* = 0.49	FE	*I*^2^ = 35%; *P* = 0.18
Hepatic flexure	6	6717/1913	1.10 [0.96, 1.27]; *P* = 0.18	FE	*I*^2^ = 12%; *P* = 0.34
Transverse colon	2	1228/248	0.79 [0.48, 1.30]; *P* = 0.35	FE	*I*^2^ = 0%; *P* = 0.47
Pathological stage					
I	9	2134/1119	0.91 [0.76, 1.11]; *P* = 0.36	FE	*I*^2^ = 0%; *P* = 0.65
II	11	2279/1256	0.98 [0.84, 1.14]; *P* = 0.75	FE	*I*^2^ = 0%; *P* = 0.79
III	11	2279/1256	0.88 [0.75, 1.02]; *P* = 0.10	FE	*I*^2^ = 46%; *P* = 0.05
IV	4	874/569	1.57 [1.07, 2.29]; *P* = 0.02	FE	*I*^2^ = 0%; *P* = 0.82
Surgical entry					
Open	2	5548/1567	1.43 [1.23, 1.66]; *P* < 0.00001	FE	*I*^2^ = 0%; *P* = 0.54
Laparoscopic	2	5548/1567	0.88 [0.78, 0.99]; *P* = 0.04	FE	*I*^2^ = 32%; *P* = 0.23
Procedure					
Right hemicolectomy	2	1821/606	0.55 [0.41, 0.75]; *P* = 0.0002	FE	*I*^2^ = 47%; *P* = 0.17
Ex-right hemicolectomy	2	1821/606	1.80 [1.33, 2.45]; *P* = 0.0002	FE	*I*^2^ = 47%; *P* = 0.17
Tumor size	5	444/375	0.23 [−0.06, 0.52]; *P* = 0.12	RE	*I*^2^ = 62%; *P* = 0.03
Lymph node yield	4	141/181	1.46 [0.96, 1.96]; *P* < 0.00001	FE	*I*^2^ = 0%; *P* = 0.48
*Others*					
Readmitted within 30 days	3	6084/2007	0.90 [0.73, 1.12]; *P* = 0.36	FE	*I*^2^ = 26%; *P* = 0.26
Return to theatre	2	6019/1942	0.46 [0.16, 1.37]; *P* = 0.16	RE	*I*^2^ = 89%; *P* = 0.003

Abbreviations: *OR* odds ratio, *MD* mean difference, *Cl* confidence intervals, *DM* diabetes mellitus, *ASA* American Society of Anesthesiologists classification of physical status, *SSA* side-side anastomosis, *ESA* end-side anastomosis

### Complications

**Fig. 3:**
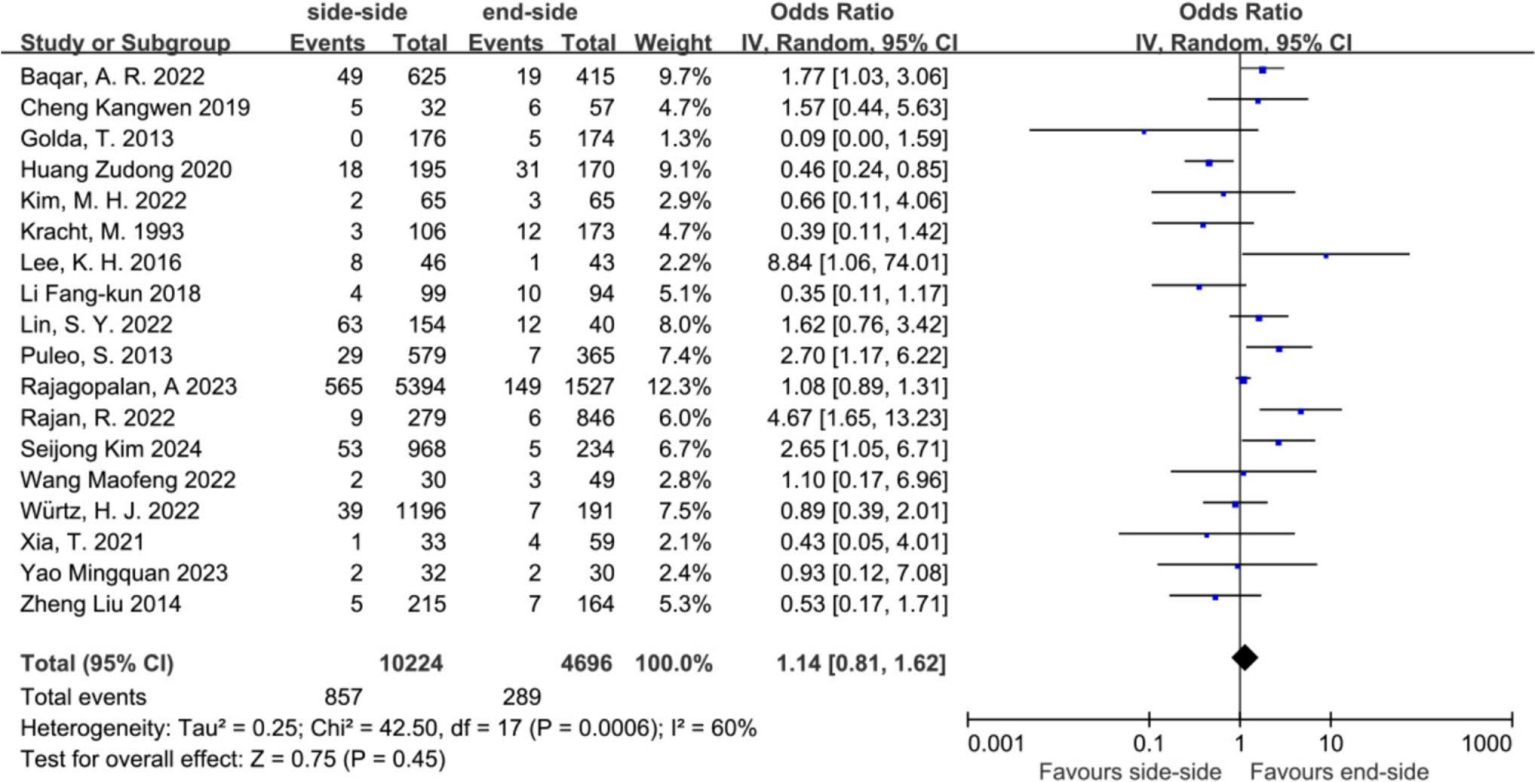
Overall anastomotic complications in the SSA versus the ESA

Data regarding overall anastomotic complications were extracted from the 18 studies. After pooling all of the data, no significance was found between the SSA and the ESA (OR = 1.14, 95% CI = 0.81 to 1.62, *P* = 0.45) (Fig. 3).

**Fig. 4:**
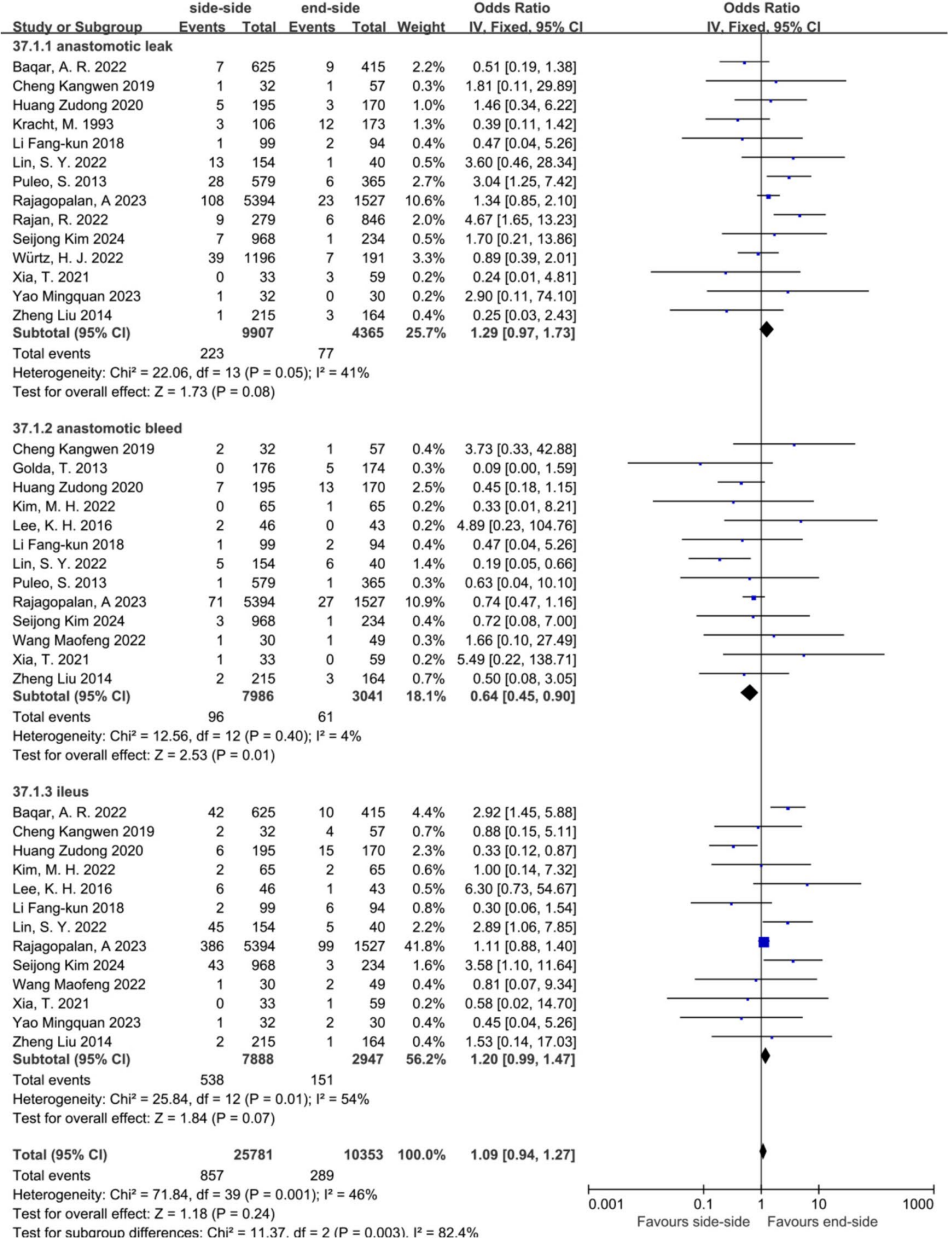
Subgroup analysis of overall anastomotic complications in the SSA versus the ESA. From top to bottom, anastomotic leakage, anastomotic bleeding and ileus

To analyze differences between various anastomotic complications in different anastomosis modalities, we performed subgroup analyses. The results showed that SSA was better than ESA in reducing anastomotic bleeding (OR = 0.64, 95% CI = 0.45 to 0.90, *P* = 0.01). In anastomotic leakage and intestinal obstruction, ESA has a significant advantage, but there was no statistical difference (OR = 1.29, 95% CI = 0.97 to 1.73, *P* = 0.08) (OR = 1.20, 95% CI = 0.99 to 1.47, *P* = 0.07) (Fig. 4).

**Fig. 5:**
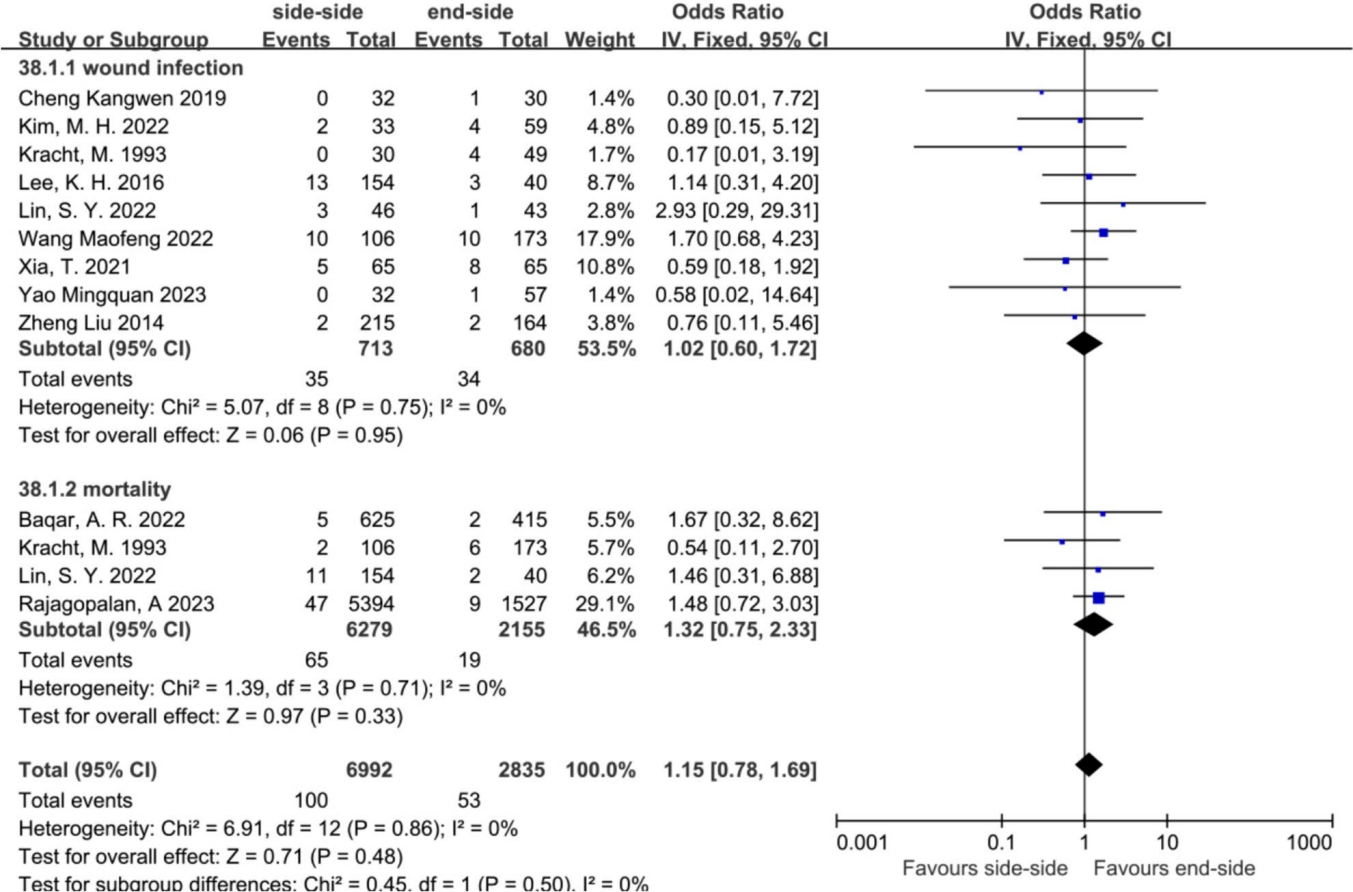
Subgroup analysis of non-anastomotic complications in the SSA versus the ESA. From top to bottom, wound infection and mortality

Non-anastomotic complications showed no significant differences, including wound infection (OR = 1.02, 95% CI = 0.60 to 1.72, *P* = 0.95) and mortality (OR = 1.32, 95% CI = 0.75 to 2.33, *P* = 0.33) (Fig. 5).

### Other outcomes

**Fig. 6:**
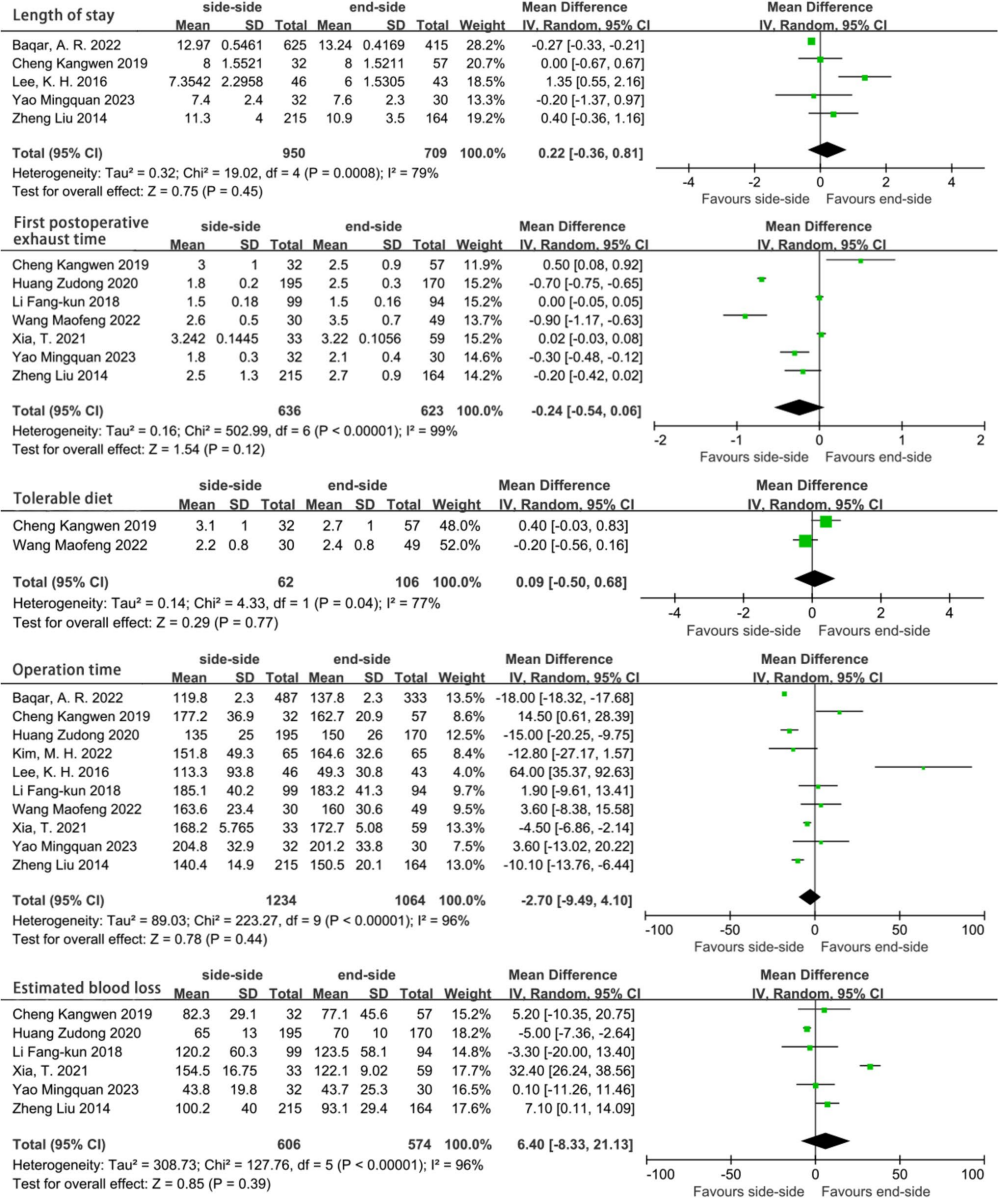
Secondary outcome subgroup analysis

Other surgical outcomes and postoperative recovery indicators were also compared between the two groups. Compared with ESA, the first postoperative exhaust time was faster in the SSA group, but it was not statistically significant (OR =  − 0.24, 95% CI =  − 0.54 to 0.06, *P* = 0.12). There was also no significant advantage in other indicators of postoperative recovery: length of hospital stay (OR = 0.22, 95% CI =  − 0.36 to 0.81, *P* = 0.45); time to first meal (OR = 0.09, 95% CI =  − 0.50 to 0.68, *P* = 0.77); there was no significant difference between ESA and SSA in terms of operation time (OR =  − 2.70, 95% CI =  − 9.49 to 4.10, *P* = 0.44) and intraoperative blood loss (OR = 6.40, 95% CI =  − 8.33 to 21.13, *P* = 0.39) (Fig. 6).

### Sensitivity analysis and publication bias

**Fig. 7:**
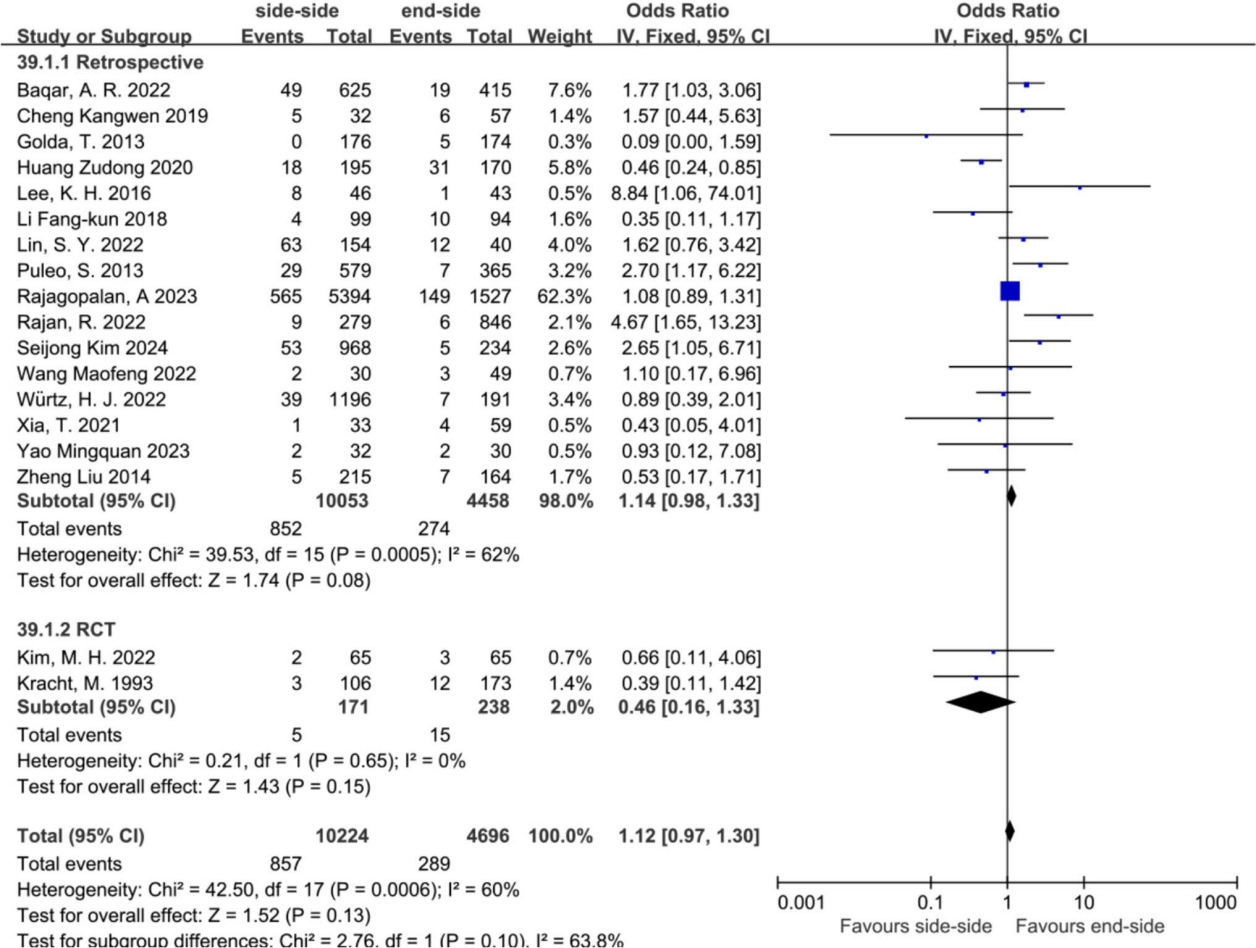
Subgroup analysis of study type in the SSA versus the ESA

**Fig. 8:**
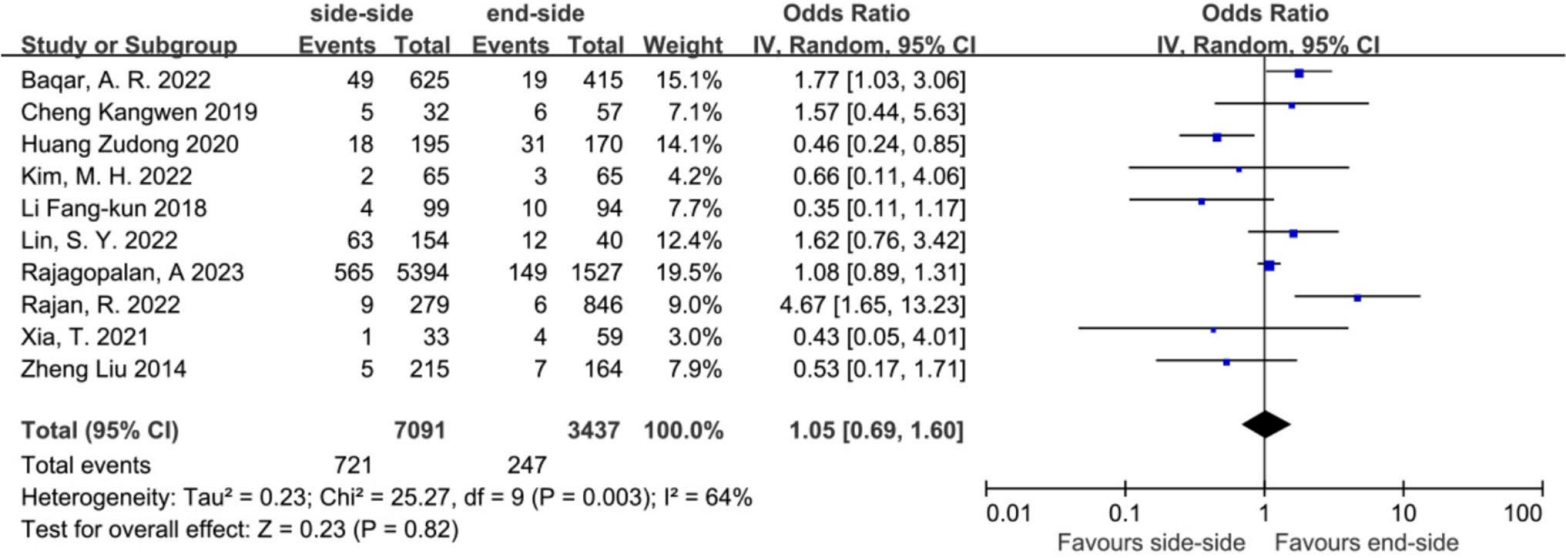
Subgroup analysis of overall anastomotic complications of SSA and ESA in ECA

**Fig. 9:**
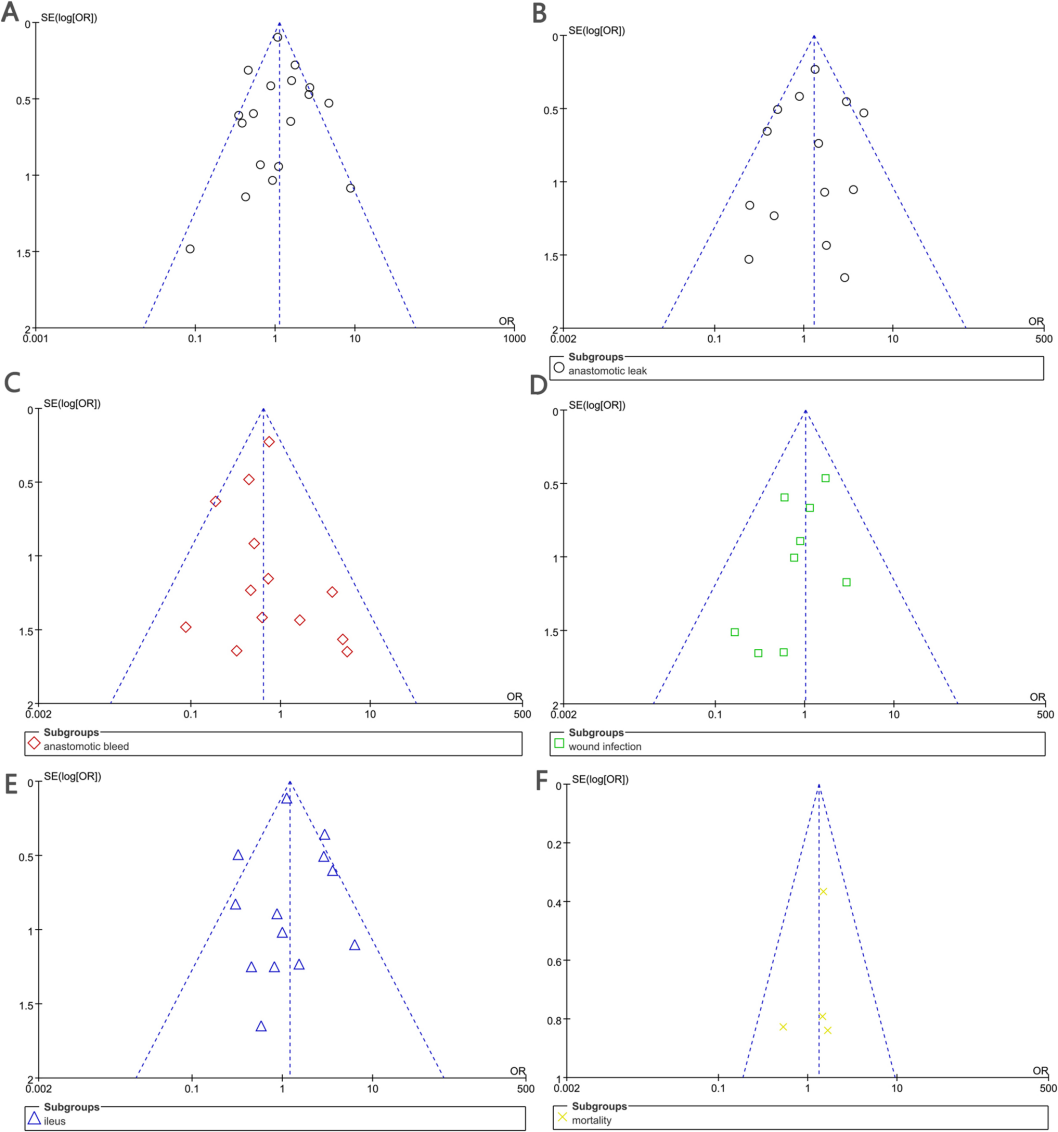
Funnel plot of complications. **A** Overall complications. **B** Anastomotic leakage. **C** Anastomotic bleeding. **D** Anastomotic infection. **E** Intestinal obstruction. **F** Mortality

To enhance the robustness of our conclusions, we conducted subgroup analyses based on study type (cohort studies and RCTs). The results indicated no significant differences in postoperative anastomotic complications in either RCTs or cohort studies (Fig. 7). In subgroup analyses of ECA, there was no statistically significant difference between SSA and ESA (Fig. 8). Publication bias for the included studies was based on a visual inspection of the funnel plot. The funnel plot was symmetrical, and no obvious publication bias was found (Fig. 9).

## Discussion

The meta-analysis included 14,555 patients from 18 studies. We found no significant difference between SSA and ESA in terms of overall postoperative anastomotic complications, non-anastomotic complications, short-term prognosis, and surgical status. However, SSA was more advantageous in reducing the incidence of postoperative anastomotic bleeding, while ESA appeared more favorable for reducing anastomotic leakage and intestinal obstruction, although these differences were not statistically significant.

Current research regarding the choice between the two anastomosis techniques remains controversial. A single-center RCT conducted in South Korea, which included 130 patients, compared the outcomes of SSA and ESA [5]. The results indicated no statistically significant differences between the two techniques in terms of postoperative hospital stay, 30-day overall complication rates, 30-day readmission rates, or failure of enhanced recovery after surgery (ERAS) protocols. In general, the choice between SSA and ESA did not significantly impact short-term postoperative outcomes. However, a 2022 systematic review, which included five studies encompassing 1986 patients, reported that ESA was associated with a lower incidence of postoperative bowel obstruction and a shorter hospital stay compared to SSA [14]. Furthermore, a retrospective cohort study by Baqar et al., analyzing 1040 patients, found that SSA significantly reduced operative time compared to ESA, but was also associated with a higher incidence of postoperative bowel obstruction [17]. These findings suggest that a more comprehensive analysis of the prognosis of the two surgical anastomosis techniques is needed.

In our study, we found that ESA is more prone to bleeding, which aligns with the characteristics of this anastomosis. In ESA, the tissue is affected by the intestinal wall, which can lead to higher anastomotic tension and postoperative bleeding. This conclusion is consistent with the findings by Golda et al. [15]. However, for intestinal obstruction, ESA appeared more advantageous, although the results were not statistically significant. This contrasts with our initial expectation, where we anticipated a higher risk of postoperative obstruction with ESA. The diameter of the end-side stapler is usually only 25–31 mm, and in cases of edema or scarring, it can easily lead to anastomotic stenosis. The small intestine and transverse colon are more prone to torsion, leading to obstruction. In contrast, the side-to-side stapler typically measures 60 mm or more. Our findings suggest that ESA may be more consistent with functional anastomosis. During intestinal peristalsis, the simultaneous contraction and relaxation of the longitudinal and circular muscles in the intestinal wall help propel contents downward along the colon [24]. This results in less damage to the ileum in ESA, leading to a lower rate of intestinal obstruction [25, 26].

After right hemicolectomy, there are several methods for intestinal reconstruction, but the classic ESA and SSA remain the most commonly used anastomotic techniques in clinical practice [27]. Regarding the surgical approach, the procedure can be performed either inside or outside the body [28]. The ESA is typically performed outside the body, mainly due to the flexibility, spatial constraints, and technical complexity of laparoscopic instruments [29, 30]. On the other hand, the SSA is generally performed inside the body to ensure precise anastomosis and minimize trauma, while considering the limitations of laparoscopic surgery and the natural anatomical positioning of the bowel [29]. Not only is the in-body anastomosis technically feasible, but it also helps reduce the risk of postoperative complications [31]. However, due to the surgeon’s experience and preference, most of the included studies were conducted extracorporeally.

Currently, there is no clear consensus on the choice between end-to-side anastomosis (ESA) and side-to-side anastomosis (SSA). However, as we move into the era of minimally invasive surgery, particularly with techniques like NOTES (Natural Orifice Transluminal Endoscopic Surgery), where the abdominal wall is not opened and the bowel is not exteriorized, SSA might be easier to perform. This approach could potentially simplify the procedure while helping to reduce anastomotic bleeding without significantly increasing the risk of postoperative complications.

A previous meta-analysis synthesized the differences between the two anastomosis, but the feasibility of the results was low due to the small number of included studies [14]; in recent years, with the increasing number of studies conducted, it is necessary to comprehensively evaluate the advantages and disadvantages of each of the two surgical modalities. Therefore, we comprehensively analyzed the relationship between the two anastomosis modalities and postoperative complications.

However, this meta-analysis has several limitations. First, only two randomized controlled trials were included, which may introduce bias and reduce the reliability of the conclusions. Second, due to limited data, variations in stapler and suture types—including size, brand, and whether the anastomosis was fully laparoscopic or laparoscopic-assisted—resulted in high heterogeneity among studies, particularly in terms of postoperative recovery. Third, the scarcity of available data prevented an analysis of long-term patient prognosis.

In addition, one potential limitation of this study is the limited data on intracorporeal anastomosis (ICA). However, since most studies performed SSA and ESA using ECA, a subgroup analysis of ECA cases was conducted, which revealed no significant difference in anastomotic complications between SSA and ESA. This suggests that the observed outcomes were primarily influenced by the anastomotic technique itself rather than the anastomotic approach (ECA vs. ICA). Future studies with more intracorporeal anastomosis (ICA) cases are needed to further validate these findings.

Both techniques have their advantages, and the choice of anastomosis should be based on the surgeon’s experience, intraoperative anatomical considerations, and patient-specific factors. SSA can reduce anastomotic bleeding. In contrast, ESA may be able to reduce postoperative obstruction or anastomotic leakage, but further research is needed. In light of these findings, it is recommended that surgeons choose anastomosis techniques based on individual patient anatomy, intestinal condition, and their own surgical expertise.

## Conclusion

Current clinical evidence suggests that in right hemicolectomy for cancer, SSA is more effective than ESA in reducing the incidence of postoperative anastomotic bleeding. However, no significant differences have been observed between the two techniques in terms of overall anastomotic complications, non-anastomotic complications, or short-term outcomes. Therefore, surgeons are advised to choose the appropriate anastomotic technique based on their experience and technical preference.

## Supplementary Information

Below is the link to the electronic supplementary material.

Supplementary file1 (DOCX 32 KB)

Supplementary file2 (DOCX 26 KB)

## Data Availability

No datasets were generated or analysed during the current study.

## References

[1] Guillou PJ, Quirke P, Thorpe H et al (2005) Short-term endpoints of conventional versus laparoscopic-assisted surgery in patients with colorectal cancer (MRC CLASICC trial): multicentre, randomised controlled trial. Lancet (London, England) 365:1718–1726. 10.1016/s0140-6736(05)66545-2 15894098 10.1016/S0140-6736(05)66545-2

[2] Buunen M, Veldkamp R, Hop WC et al (2009) Survival after laparoscopic surgery versus open surgery for colon cancer: long-term outcome of a randomised clinical trial. Lancet Oncol 10:44–52. 10.1016/s1470-2045(08)70310-3 19071061 10.1016/S1470-2045(08)70310-3

[3] Funariu G, Pop CE, Ionescu C, Scurtu R, Dindelegan G (2001) Stapled anastomoses in colorectal surgery. Chirurgia (Bucharest, Romania: 1990) 96:213–219 12731158

[4] Kracht M, Hay JM, Fagniez PL, Fingerhut A (1993) Ileocolonic anastomosis after right hemicolectomy for carcinoma: stapled or hand-sewn? A prospective, multicenter, randomized trial. Int J Colorectal Dis 8:29–33. 10.1007/bf00341273 8492040 10.1007/BF00341273

[5] Kim MH, Kang SI, Cho JR et al (2022) Objective recovery time with end-to-side versus side-to-side anastomosis after laparoscopic right hemicolectomy for colon cancer: a randomized controlled trial. Surg Endosc 36:2499–2506. 10.1007/s00464-021-08536-5 34008107 10.1007/s00464-021-08536-5

[6] Liu Z, Wang G, Yang M et al (2014) Ileocolonic anastomosis after right hemicolectomy for colon cancer: functional end-to-end or end-to-side? World J Surg Oncol 12:306. 10.1186/1477-7819-12-306 25287418 10.1186/1477-7819-12-306 PMC4198793

[7] Lee KH, Lee SM, Oh HK et al (2016) Comparison of anastomotic configuration after laparoscopic right hemicolectomy under enhanced recovery program: side-to-side versus end-to-side anastomosis. Surg Endosc 30:1952–1957. 10.1007/s00464-015-4420-6 26198156 10.1007/s00464-015-4420-6

[8] Puleo S, Sofia M, Trovato MA et al (2013) Ileocolonic anastomosis: preferred techniques in 999 patients. A multicentric study. Surg Today 43:1145–1149. 10.1007/s00595-012-0381-8 23111464 10.1007/s00595-012-0381-8

[9] Zudong H, Dingming L, Xiaoping L, Yanning L, Jun Z (2020) A comparative study of clinical effect of different anastomosis methods in laparoscopic assisted right hemicolectomy. J Dig Oncol 12(2):147–150

[10] Hozo SP, Djulbegovic B, Hozo I (2005) Estimating the mean and variance from the median, range, and the size of a sample. BMC Med Res Methodol 5:13. 10.1186/1471-2288-5-13 15840177 10.1186/1471-2288-5-13 PMC1097734

[11] Wan X, Wang W, Liu J, Tong T (2014) Estimating the sample mean and standard deviation from the sample size, median, range and/or interquartile range. BMC Med Res Methodol 14:135. 10.1186/1471-2288-14-135 25524443 10.1186/1471-2288-14-135 PMC4383202

[12] Xia T, Pan Z, Zhang J, Xu G (2021) Comparison of postoperative recovery of patients who underwent laparoscopic-assisted radical resection of right colon cancer with modified triangular anastomosis or tubular anastomosis: a retrospective cohort study. BMC Surg 21:77. 10.1186/s12893-021-01086-6 33568123 10.1186/s12893-021-01086-6 PMC7877016

[13] Rajagopalan A, Centauri S, Antoniou E et al (2023) Right hemicolectomy for colon cancer: does the anastomotic configuration affect short-term outcomes? ANZ J Surg 93:1870–1876. 10.1111/ans.18523 37259620 10.1111/ans.18523

[14] Lin SY, Liang Buan BJ, Sim W et al (2022) Side-to-side versus end-to-side ileocolic anastomosis in right-sided colectomies: a cohort control study. J Minimal Access Surg 18:408–414. 10.4103/jmas.jmas_161_21 10.4103/jmas.jmas_161_21 PMC9306133 35046183

[15] Golda T, Zerpa C, Kreisler E, Trenti L, Biondo S (2013) Incidence and management of anastomotic bleeding after ileocolic anastomosis. Colorectal Dis 15:1301–1308. 10.1111/codi.12309 23710632 10.1111/codi.12309

[16] Würtz HJ, Bundgaard L, Rahr HB, Frostberg E (2022) Anastomosis technique and leakage rates in minimally invasive surgery for right-sided colon cancer. A retrospective national cohort study. Int J Colorectal Dis 37:701–708. 10.1007/s00384-022-04107-9 35150297 10.1007/s00384-022-04107-9

[17] Rajan R, Arachchi A, Metlapalli M et al (2022) Ileocolic anastomosis after right hemicolectomy: stapled end-to-side, stapled side-to-side, or handsewn? Int J Colorectal Dis 37:673–681. 10.1007/s00384-022-04102-0 35124716 10.1007/s00384-022-04102-0

[18] Min-quan Y, Yu-peng J, Da-zhuang S (2023) The use of overlap anastomosis in complete laparoscopic radical right hemicolectomy. Zhejiang Clin Med 25(12):1741–1743

[19] Mao-feng W, Hui L, Lei W (2022) Reconstruction of digestive tract after laparoscopic right hemicolectomy. Chin J Surg Oncol 14(5):489–493

[20] Kim S, Huh JW, Lee WY et al (2024) Comparative analysis of functional end-to-end and end-to-side anastomosis in laparoscopic right hemicolectomy for colon cancer. Surgery 180:109051. 10.1016/j.surg.2024.109051 39740601 10.1016/j.surg.2024.109051

[21] Kang-wen C, Gui-he W, Kuan-shan S (2019) A retrospective controlled study of two mechanical anastomosis modalities in laparoscopy-assisted surgery for right colon cancer. Chinese J Bases Clin Gen Surg 26(7):856–860. 10.7507/1007-9424.201902031

[22] Fang-kun L, Xiu-tao C, Huichen X (2018) Comparison of different anastomosis methods in laparoscopic assisted right hemicolectomy. J Laparosc Surg 23(10):782–785

[23] Baqar AR, Wilkins S, Wang WC et al (2022) A comparison of extracorporeal side to side or end to side anastomosis following a laparoscopic right hemicolectomy for colon cancer. ANZ J Surg 92:1472–1479. 10.1111/ans.17701 35403808 10.1111/ans.17701 PMC9324090

[24] Smith TK, Robertson WJ (1998) Synchronous movements of the longitudinal and circular muscle during peristalsis in the isolated guinea-pig distal colon. J Physiol 506(Pt 2):563–577. 10.1111/j.1469-7793.1998.563bw.x 9490879 10.1111/j.1469-7793.1998.563bw.x PMC2230717

[25] Ibáñez N, Abrisqueta J, Luján J et al (2019) Isoperistaltic versus antiperistaltic ileocolic anastomosis. Does it really matter? Results from a randomised clinical trial (ISOVANTI). Surg Endosc 33:2850–2857. 10.1007/s00464-018-6580-7 30426254 10.1007/s00464-018-6580-7

[26] Sciuto A, Merola G, De Palma GD et al (2018) Predictive factors for anastomotic leakage after laparoscopic colorectal surgery. World J Gastroenterol 24:2247–2260. 10.3748/wjg.v24.i21.2247 29881234 10.3748/wjg.v24.i21.2247 PMC5989239

[27] Zhao LY, Chi P, Ding WX et al (2014) Laparoscopic vs open extended right hemicolectomy for colon cancer. World J Gastroenterol 20:7926–7932. 10.3748/wjg.v20.i24.7926 24976728 10.3748/wjg.v20.i24.7926 PMC4069319

[28] Perivoliotis K, Tzovaras G, Tepetes K, Baloyiannis I (2024) Comparison of intracorporeal and extracorporeal anastomosis in laparoscopic right colectomy: an updated meta-analysis and trial sequential analysis. Updat Surg 76:375–396. 10.1007/s13304-023-01737-8 10.1007/s13304-023-01737-8 38216794

[29] Ricci C, Casadei R, Alagna V et al (2017) A critical and comprehensive systematic review and meta-analysis of studies comparing intracorporeal and extracorporeal anastomosis in laparoscopic right hemicolectomy. Langenbecks Arch Surg 402:417–427. 10.1007/s00423-016-1509-x 27595589 10.1007/s00423-016-1509-x

[30] Wang X, Ni H, Jia W et al (2022) Value of different anastomoses in laparoscopic radical right hemicolectomy for right-sided colon cancer: retrospective study and literature review. World J Surg Oncol 20:318. 10.1186/s12957-022-02789-7 36171623 10.1186/s12957-022-02789-7 PMC9520856

[31] Meyer J, Wijsman J, Crolla R, van der Schelling G (2023) Implementation of totally robotic right hemicolectomy: lessons learned from a prospective cohort. J Robot Surg 17:2315–2321. 10.1007/s11701-023-01646-3 37341877 10.1007/s11701-023-01646-3 PMC10492732

