# Supplementary material for: Comparison study of two anastomosis techniques in right hemicolectomy: a systematic review and pooling up analysis
Source: Int J Colorectal Dis. 2025 Feb 25;40(1):50. doi: 10.1007/s00384-025-04835-8 (PMC11850514; doi:10.1007/s00384-025-04835-8)
Supplement: Supplementary file 2 — Supplementary file2 (DOCX 26 KB) [file 384_2025_4835_MOESM2_ESM.docx]

**Article type**: Original article.

**Title**: Comparison study of two anastomosis techniques in right hemicolectomy: a systematic review and pooling up analysis.

**Author**: Xiao-Qiang Zhang, MD^1^, Run-xi Tang^2^, Chao-Fu Zhang, MD^1^, Ming-Yang Xia, MD^1^, Lei-Yuan Shuai, MD^3^, Hua Tang, MD, PhD^1^, Guang-Yan Ji, MD, PhD^1^*

1^#^ Department of Gastrointestinal Surgery, the First Affiliated Hospital of Chongqing Medical University, Chongqing 400016, China.

2^#^ Shanxi provincial Institute of Traditional Chinese Medicine, Shanxi 030021, China.

3^#^ Department of Anorectal Surgery, Jiangjin Central Hospital of Chongqing, Chongqing 404000, China.

**Corresponding Author:** Guang-Yan Ji, MD, PhD, Department of Gastrointestinal Surgery, The First Affiliated Hospital of Chongqing Medical University, No.1 Youyi Road, Yuanjiagang District, Chongqing 400016, China.

**E-mail address:** [jiguangyan168@163.com](mailto:jiguangyan168@163.com)

**Supplementary Materials - Index**

[NOS Criteria and Scoring Results 2](#_Toc183636849)

# NOS Criteria and Scoring Results

1. Representativeness of the exposed cohort
2. Truly representative of the general population 
3. Somewhat representative of the general population 
4. Selected group of users e.g. nurses, volunteers, hospitalized individuals, with specific health condition
5. No description of the derivation of the cohort
6. Selection of the non-exposed cohort
7. Drawn from the same community as the exposed cohort 
8. Drawn from a different source
9. No description of the derivation of the non-exposed cohort
10. Ascertainment of exposure
11. Secure record (i.e. medical records incl. lab results, or blood measures) 
12. Structured interview or hospital/insurance data with no external validation
13. Written self-report
14. No description
15. Demonstration that outcome of interest was not present at start of study/before ascertainment of exposure
16. Yes, if individuals with outcome of interest diagnosed/reported within the first year after study entry/ascertainment of exposure are excluded or considered as non-exposed 
17. No, otherwise

**Comparability (x/2)**

1. Comparability of cohorts on the basis of the design or analysis

a) Study controls for age, sex, follow-up time, calendar year (if overall study period≥15 years), and major site-specific factors, i.e. smoking for lung cancer; parity, first-degree family history, HRT/menopausal status for breast cancer; parity, oral contraception (ovarian cancer); 1st-degree family history, ethnicity for prostate cancer; calendar year for thyroid cancer

b) Study controls for any additional factor (e.g. ethnicity, socioeconomic/educational status, other family history of cancer, breastfeeding, other reproductive factors, alcohol consumption, diet, physical activity, body-mass index/obesity, diabetes, etc.) 

**Outcome (x/3)**

1. Assessment of outcome
2. Independent blind (e.g. national/regional cancer registry) assessment (including radiology/histology/anapath. confirmation)
3. Record linkage (hospital/insurance data with no external validation, i.e. radiology/histology/anapath.)
4. Self-report
5. No description
6. Was follow-up long enough for outcomes to occur
7. Yes, if mean/median follow-up time≥5 years 
8. No, if mean/median follow-up time <5 years, or not stated
9. Adequacy of follow up of cohorts
10. Complete follow up - all subjects accounted for 
11. Subjects lost to follow up unlikely to introduce bias - small number lost≥90% follow up, or description provided of those lost, or linkage with national registry (emigration and linkage failure rate assumed to be <10%) 
12. Follow up rate <90% and no description of those lost, or suspicion that loss-of-follow-up is related to the outcome
13. No statement

| AUTHOR | 1 | 2 | 3 | 4 | 5 | 6 | 7 | 8 | TOTAL |
| --- | --- | --- | --- | --- | --- | --- | --- | --- | --- |
| *Rajagopalan, A 2023* | a | a | a | a | a | b | b | a | 8 |
| *Würtz, H. J. 2022* | a | a | a | a | a | b | b | a | 8 |
| *Rajan, R. 2022* | a | a | a | a | a | b | b | d | 7 |
| *Baqar, A. R. 2022* | a | a | a | a | a | b | a | a | 9 |
| *Xia, T. 2021* | a | a | a | a | a | b | b | d | 7 |
| *Lin, S. Y. 2022* | a | a | a | a | a | b | b | d | 7 |
| *Lee, K. H. 2016* | a | a | a | a | a | b | b | d | 7 |
| *Puleo, S. 2013* | a | a | a | a | b | b | b | d | 6 |
| \| ***Golda, T. 2013*** \| ***2022*** \| \| --- \| --- \| | a | a | a | a | a | b | a | a | 9 |
| *Yao Mingquan 2023* | a | a | a | a | a | b | b | d | 7 |
| *Wang Maofeng 2022* | a | a | a | a | a | b | b | d | 7 |
| *Huang Zudong 2020* | a | a | a | a | a | b | b | d | 7 |
| *Cheng Kangwen 2019* | a | a | a | a | a | b | b | d | 7 |
| *Li Fang-kun 2018* | a | a | a | a | a | b | a | a | 9 |
| *Zheng liu 2014* | a | a | a | a | a | b | a | a | 9 |
| *Seijong Kim 2024* | a | a | a | a | a | b | a | a | 9 |
